# Editorial: The Lesser Known World of RNA Polymerases

**DOI:** 10.3389/fmolb.2021.811413

**Published:** 2021-12-02

**Authors:** Olga Calvo, Athar Ansari, Francisco Navarro

**Affiliations:** ^1^ Instituto de Biología Funcional y Genómica, Consejo Superior de Investigaciones Científicas, Universidad de Salamanca, Salamanca, Spain; ^2^ Department of Biological Science, Wayne State University, Detroit, MI, United States; ^3^ Departamento de Biología Experimental-Genética, Universidad de Jaén, Jaén, Spain; ^4^ Centro de Estudios Avanzados en Aceite de Oliva y Olivar, Universidad de Jaén, Jaén, Spain

**Keywords:** RNA polymerase, transcription, evolution, biogenesis, posttranslational modifications, diseases, splicing, transcription factors

**FIGURE 1:**
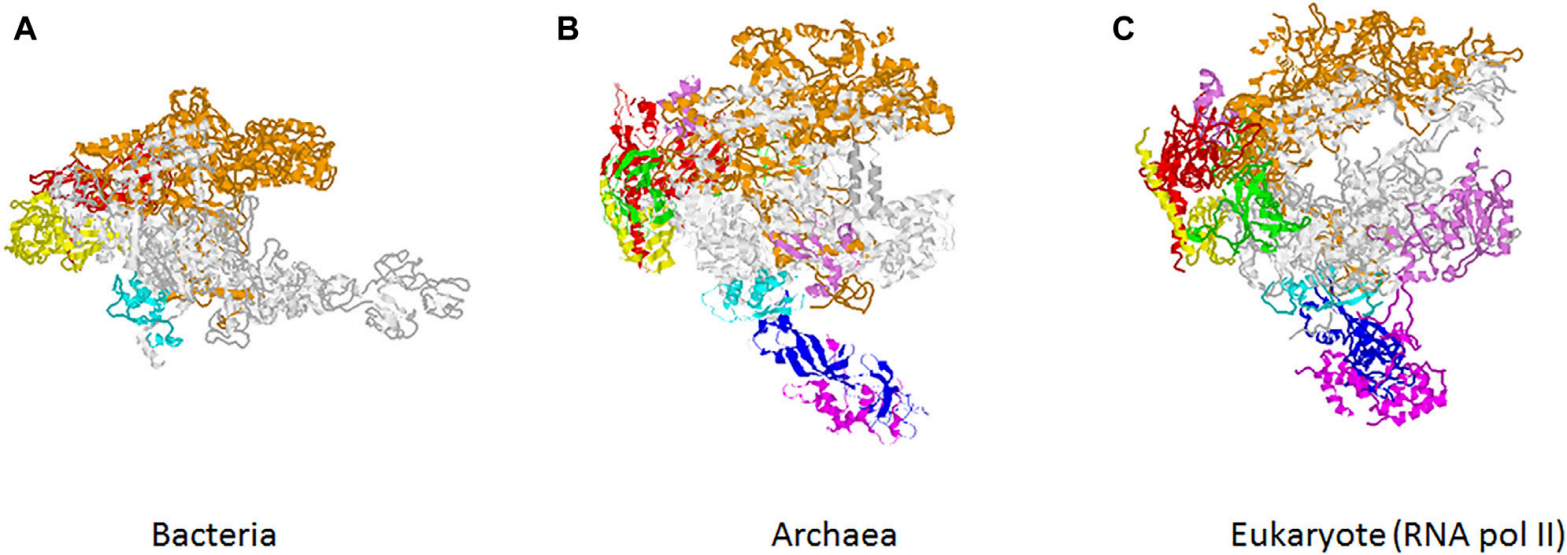
Evolution of RNA polymerases. Structure of RNA polymerase from bacteria **(A)**, archaea **(B)** and eukaryotic RNA pol II **(C)**. Color code corresponds to the previously used for *Saccharomyces cerevisiae* RNA pol II subunits (Armache et al., 2003). Similar colors are used for homologous subunits in bacterial and archaeal RNA pols.

According to the central dogma of molecular biology, genetic information is passed from DNA to RNA to protein. The transmission of information from DNA to RNA is called transcription and is carried out by the RNA polymerases (RNA pols). The RNA pols from bacteria to eukaryotes are multimeric enzymes which show a high degree of conservation in terms of their structure and functionality (Cramer, 2019; Werner and Grohmann 2011) (Figure 1). Notably, while bacteria and archaea contain only one RNA pol, most eukaryotes contain three different enzymes, RNA pol I, II and III, with the exception of plants that also have two additional RNA pols, IV and V, which evolved from RNA pol II (Ream et al., 2014). RNA pol I synthesizes the precursor of the three largest rRNAs, RNA pol III produces mostly tRNAs, the 5S rRNA and several short non-translated RNAs and RNA pol II give rise to all mRNAs and many non-coding RNAs, including miRNA. Finally, RNA pol IV and V participate in transcriptional silencing and also in production of non-coding RNAs involved in the development and response to environmental changes (Werner and Grohmann 2011; Ream et al., 2014; Cramer, 2019). The enzyme exhibits a broad evolutionary diversity, functional dynamism and pleiotropic role in biological systems. However, many aspects of RNA pols, including their biogenesis, function, and even their impact in different cellular processes or health, have not been deeply investigated. This special issue is an attempt to cover some of the lesser-known aspects of RNA pol diversity, dynamism, function and evolutionary conservation. In addition, this issue also considers transcription factors as part of the transcriptional machinery.

Despite variation in structure and subunit composition, RNA pols from different organisms harbors conserved features (Lane and Darst, 2010a; Lane and Darst, 2010b). The article by Lei and Burton compares the three-dimensional structure of archaeal, bacterial and eukaryotic RNA pols. Their analyses revealed that the enzyme, in all three domains of life, are of two-double-ψ-β-barrel (2-DPBB) type. In addition to 2-DPBB, the catalytic core of multisubunit RNA pols is comprised of a conserved bridge helix and trigger loop. Lei and Burton propose that the 2-DPBB family of multisubunit RNA pols might have evolved prior to the last universal common cellular ancestor during evolution.

Nanoarchaea is a highly diverged archaeal phylum with many unusual biological features. Nottebaum and Weinzierl describe that several of the key motifs in the active center of *Nanoarchaeum equitans* RNA pol contain unusual and radical substitutions expected to be harmful to the catalytic activity. However, the authors reconstituted a RNA pol complex *in vitro* with transcription activity, concluding that sequence changes do not adversely affect catalytic activity, even if they are unusual and localized in key motifs. Moreover, they identified a stringent atypical requirement for fluoride ions for maximal RNA pol activity, proposing a model where more “conventional” archea will not use it.

The work by Barba-Aliaga et al. focuses on nuclear RNA pols in eukaryotes and summarizes their evolutionary origin and functional reasons that could have led to their multiplicity. Furthermore, authors discuss the regulation and the homeostasis of the different RNA products that they synthesise. The authors present several studies to show how the coordination between RNA pols activities is necessary for cellular processes, such as the influence of RNA pols on the translation machinery synthesis (ribosomes and tRNAs) or how eukaryotic RNA pols transcription regulation occurs with respect to the changes in cellular volume.

Plants are the only known eukaryotic organisms containing two addition RNA pols (IV and V), in addition to RNA pol I-III, which evolved from RNA pol II (Ream et al., 2013). The work by Fernández-Parras et al., investigates the transcriptional regulation of the RNA pols common subunits genes in olive tree cultivar (*Olea europaea* L. cv. Picual) and shows that they are spatio-temporally regulated, as well as regulated by biotic and abiotic stresses. This work opens questions about the existence of multiple RNA pols variants in polyploid organisms.

A model for the biogenesis of eukaryotic RNA pol II has been proposed based on the bacterial RNA pol formation, a sequential process involving participation of several subassembly complexes that leads to the complete enzyme assembly in the cytoplasm before its nuclear import (Wild and Cramer, 2012). However, despite recent progress, the assembly of RNA pols remains poorly described. The work by Garrido-Godino et al., focuses on the knowledge of biogenesis of RNA pols in yeast. The authors review the mechanisms and proteins (assembly and transport factors) involved in these processes and make the comparison with human factors described previously. In addition, the manuscript by Turowski and Boguta summarizes the current knowledge on the biogenesis of the RNA pol I and III and focuses on the model of their co-translational assembly, based on recent publications, showing the importance of Rpb10, Rpc19 and Rpc40, and of the Rbs1 protein in the assembly of RNA pol I and III complexes.

Notably, mutations of RNA pols lead to diseases and disorders. Some of these are suggested to be associated with RNA pol III assembly. Although the causal relationship between RNA pols mutations and disease development is widely accepted, the associated molecular mechanisms are poorly understood. The work by Lata et al. reviews the current knowledge regarding the functional impact of specific mutations, possible Pol III-related disease-causing mechanisms, and animal models that may help to better understand the links between Pol III mutations and disease. Similarly, a large number of genetic diseases associated with RNA pol I mutations exists. They are collectively called ribosomopathies. The understanding of the precise mechanistic of Pol I transcription opens broad perspectives in health-related research areas.

Azouzi et al. nicely review recent advances in the field of RNA pol I transcription elongation, revealed using nucleotide resolution techniques. These advances showed the connection between the production of rRNA and nascent rRNA folding. Indeed, rRNA folding during transcription seems to be an anti-pausing mechanism favoring transcription elongation because rRNA secondary structures prevent backtracking. Furthermore, they also discuss mechanisms involved in RNA pol I termination. Based on recent discoveries by Darrière et al. (2019), using a super-active RNA polI mutant, the authors propose that premature transcription termination at defined positions can control rRNA production *in vivo*.

There is an enormous interest in deciphering how RNA pols integrate the information that cells receive and how RNA pols are coordinated and communicated to regulate gene expression in response to physiological and pathological conditions. In this regard, Delgado-Román and Muñóz-Centeno proposed that RNA pols I and III activities should be connected to regulate cell cycle progression. How cell cycle regulation is affected by the balance between the three RNA pols products and RNA pols assembly is discussed. The authors focus on ribogenesis, a process that requires the activity of all three RNA pols (de la Cruz et al., 2018), and discuss how the balanced production of ribosomal components prevents G1 arrest in budding yeast and mammalian cells, which show strong analogies (Bursac et al., 2012; Gómez-Herreros et al., 2013).

González-Jiménez et al. propose that phosphorylation may have a role in the coordination of the three transcription machineries. Various studies have reported that several subunits of RNA pol I, II and III are susceptible to phosphorylation (for instance, Šoštarić, et al., 2018; Lanz et al., 2021). Some of these phosphorylation sites are distributed within subunits common to all three RNA pols. This suggests that phosphorylation events might finely modulate the activities of all RNA pols and give rise to the speculation that they can play a crucial regulatory role in the coordination between the three RNA pols, which, so far, has not been investigated enough. In this review the authors compile all the known phosphorylation sites identified for the three RNA pols, localized most of them within the respective complexes and discussed their possible roles. This is a valuable information for researchers interested in this exciting and promising field of study.

Since their discovery, the biological significance of introns in the eukaryotic genome has remained an enigma. A number of studies in a diversity of eukaryotes have revealed that the process of splicing, which removes an intron from a primary transcript, is often a positive regulator of transcription (Gallegos and Rose 2015; Shaul 2017). The article by Dwyer et al. proposes a novel mechanism of splicing-mediated regulation of transcription by RNA pol II through modulating the gene architecture. The transition in topology of a gene from linear to a loop during cotranscriptional splicing and the mechanism of enhancement of transcription by the looped structure is being discussed.

Transcription by RNA pol II in eukaryotes is facilitated by a number of transcription factors. TFIIB is one such essential general transcription factors (Deng and Roberts 2007). The article by O’ Brien and Ansari focuses on a rather unexpected role of TFIIB during viral pathogenesis. The article describes in detail the targeting of TFIIB by viral transcriptional regulators during pathogenesis. Likely reasons for preferred targeting of TFIIB over other general transcription factors by viruses are discussed. This makes TFIIB a potential target of antiviral therapies.

TFII-I is another transcription factor of RNA pol II (Roy 2012). It was originally discovered as an initiator-binding protein that helps in initiation of transcription from TATA-less promoters. Further research revealed that TFII-I is involved in post-initiation steps as well. Linzer et al. discuss multiple aspects of TFII-I participation in the transcription cycle. In addition to affecting initiation from a subset of promoters, TFII-I is involved in transcription elongation by regulating pausing of RNA pol. The involvement of this factor in cancer, neurological and immunological disorders in humans, development in mice, and induction of pluripotency is discussed.

It was known since a long time that the transcriptionally active UV-damaged regions of genome are repaired more efficiently than the non-transcribed regions. The factor responsible for the transcription-coupled repair of damaged DNA in prokaryotes is *mfd* (Selby and Sancar, 1993). The article by Lindsey-Boltz and Sancar discusses the recent advances in three-dimensional structure and single molecule studies pertaining to *mfd*. These studies have revealed that *Mfd* binds stalled RNA pols even in the absence of UV damage and helps the pol operate in hard-to-transcribe regions. The possibility of *mfd*-RNA polymerase interaction contributing to both, promotion and prevention of mutagenesis in a context-dependent manner, is discussed.

The articles in this special issue cover some, but not all, lesser-known aspects of RNA pols and highlight that many mechanistic, structural and/or evolutionary aspects of RNA pols, among others, remain unexplored or are still not well investigated. Futures investigations on RNA pols will greatly help to understand gene expression regulation, where transcription is the bottle neck.
